# National cultural characteristics as defined by Hofstede’s dimensions and their associations with population health

**DOI:** 10.1186/s12889-025-22915-w

**Published:** 2025-05-22

**Authors:** Radka Zidkova, Jana Furstova, Klara Malinakova, Jitse P. van Dijk, Peter Tavel, Gabriel Gulis

**Affiliations:** 1 OUSHI – Olomouc University Social Health Institute, Palacký University Olomouc, Univerzitni 244/22, Olomouc, 771 11 Czech Republic; 2 Department of Community and Occupational Medicine, University Medical Center Groningen, University of Groningen, Groningen, The Netherlands; 3 Graduate School Kosice Institute for Society and Health, P. J. Safarik University, Kosice, Slovak Republic; 4 Unit for Health Promotion Research, University of Southern Denmark, Esbjerg, Denmark

**Keywords:** Cultural characteristics, Hofstede’s index, Population health, Burden of disease

## Abstract

**Background:**

Understanding population health trends and their key determinants is essential for planning health services and implementing effective interventions. One of these determinants may be national cultural characteristics that are related to various health outcomes and health-related behaviours. However, little is known about their potential association to overall burden of disease. Thus, this study examined whether cultural characteristics expressed by Hofstede indexes are associated with the burden of disease.

**Methods:**

We used data from open-source databases – Hofstede’s Cultural Index, the Global Burden of Diseases (GBD) and the Human Development Index (HDI). The final sample comprised 69 countries covering all the continents. The burden of disease was measured using disability-adjusted life years (DALYs), years lived with disabilities (YLD), and years of life lost (YLL). National cultural characteristics were measured using Hofstede’s dimensions. Bayesian correlation analyses were conducted to assess the relationships between cultural dimensions and health outcomes, stratified by countries' HDI levels.

**Results:**

In countries with a very high HDI, there was strong evidence (Bayes Factor > 10) of a positive correlation of Power distance with the total disability-adjusted life years (*r* = 0.448) and years of life lost (*r* = 0.528), and Individualism (*r* = 0.667) and Indulgence (*r* = 0.494) with years lived with disabilities. In contrast, Long-term orientation negatively correlated of with years lived with disabilities (*r* = -0.527) and Indulgence with disability-adjusted life years (*r* = -0.437) and years of life lost (*r* = -0.537). Further, Power distance and Indulgence were correlated with the majority of the GBD indicators and Individualism with a few GBD indicators. In countries with a high and medium HDI, strong evidence of the associations was found in only a few cases.

**Conclusion:**

We found a correlation between national cultural characteristics and burden of disease. Policy-makers should consider integrating cultural factors into public health strategies to better align healthcare interventions with the local population’s values and behaviours. Moreover, cross-cultural research and collaboration should increase to understand how cultural influences can be used to mitigate disease burdens and improve health outcomes globally. This study also opens a potentially new research area within population health research.

## Background

Understanding trends in population health, including those of major determinants of health, is very important both due to the planning of health services, such as health promotion, disease prevention and health care, and facilitating proper interventions [1]. Population health is defined as “the health outcomes of a group of individuals, including the distribution of such outcomes within the group” [2]. Due to the variety of available health indicators, which are usually oriented toward specific health outcomes, monitoring population health trends, especially on international or global level, is not a simple task. Not all countries operate vital statistics, nor are disease-specific registries available in some countries, and often they are not internationally harmonised. Public health research responded to this situation with the development of composite indicators: the Global Burden of Disease (GBD) study supported by the WHO, and the World Bank developed the disability-adjusted life years (DALYs) indicator [3] as a composite measure comprising the impact of premature mortality (years of life lost, YLL) and the years lived with disabilities (YLD) [4]. DALYs quantify the difference between a current situation and an ideal situation in which everyone lives up to the age of the standard life expectancy in perfect health. One DALY is equivalent to the loss of one year of full health. Another composite indicator is, for example, the quality adjusted life years (QALY), which is frequently used for health economic considerations [5].

In line with the Dahlgren & Whitehead model of determinants of health [6], diverse living environments, differences in the availability of and access to health care and in living standards and socioeconomic differences all contribute to country differences in DALYs [7–9]. The upper level of the model refers to general socio-economic, cultural and environmental conditions under which people live and through that highlights the importance of the cultural characteristics of the country in which individuals live as a potential explanation for differences in health among countries [6]. Cultural factors also significantly influence individuals' health beliefs, behaviours, help-seeking patterns and healthcare use [10]. For example, cultural practices are associated with, dietary behaviour (e.g. mediterranean diet vs. american diet vs. central european diet etc.) [11], physical activity [12], preferences for traditional or alternative medicine [13, 14], perceptions of health risks [15], or with how individuals approach disease prevention or treatment [16]. As a result, these may have an impact on the health outcomes of the population [17, 18], and subsequently also on the burden of disease.

To measure national cultural characteristics, Hofstede’s national dimensions [19–21] are mostly used. Although there has been some criticism of the Hofstede concept [22, 23], his dimensions are the most frequently cited in the cross-cultural framework [19] validated, and considered the most comprehensive [24–26]. Hofstede defines national culture using six dimensions: Individualism, Power distance, Masculinity, Uncertainty avoidance, Long-term orientation and Indulgence. Specifically, Individualism represents the degree to which people in a society are integrated into groups; Power distance represents the extent to which a society accepts and expects that power is distributed unequally; Masculinity shows whether society is driven by competition and success (Masculine) or by caring for others and being modest; Uncertainty avoidance relates to the way society deals with the fact that the future can never be known; Long-term orientation describes how each society must keep certain links to its own past while dealing with the challenges of the present and the future; and Indulgence is the extent to which people try to control their desires and urges based on how they were raised [27, 28].

There have been only a few studies examining Hofstede’s dimensions in relation to the factors that may ultimately influence elements of the burden of disease. Previous research has suggested that national cultural characteristics may have an impact on obesity [17], respectively body mass index [21], and public self-consciousness, which in turn had an influence on people’s intention to eat a healthy diet [29], subjective well-being [30], depression [18], suicidality [31] or safety orientation [32]. Therefore, we believe that national cultural characteristics may also have an impact on the overall burden of disease, a relation which to our knowledge has not yet been studied in previous research.

Thus, the aim of our study is to investigate whether national cultural characteristics, as expressed by Hofstede’s dimensions, have an impact on the total burden of disease, and on the different groups of risk factors (environmental, biological and behavioural risk) affecting disability-adjusted life years, years lived with disabilities and years of life lost. After recognising the most relevant of Hofstede’s dimensions associated with the burden of disease, we analysed the correlation of those dimensions with the burden of disease caused by the most relevant groups of diseases. We expect to provide information on the relation of cultural characteristics to the burden of disease and thus open a potentially new research area within population health research. Exploring these correlations can also be used for practice. We also expect to provide information for future intervention areas in terms of cultural determinants of health.

## Methods

### Data

**Fig. 1:**
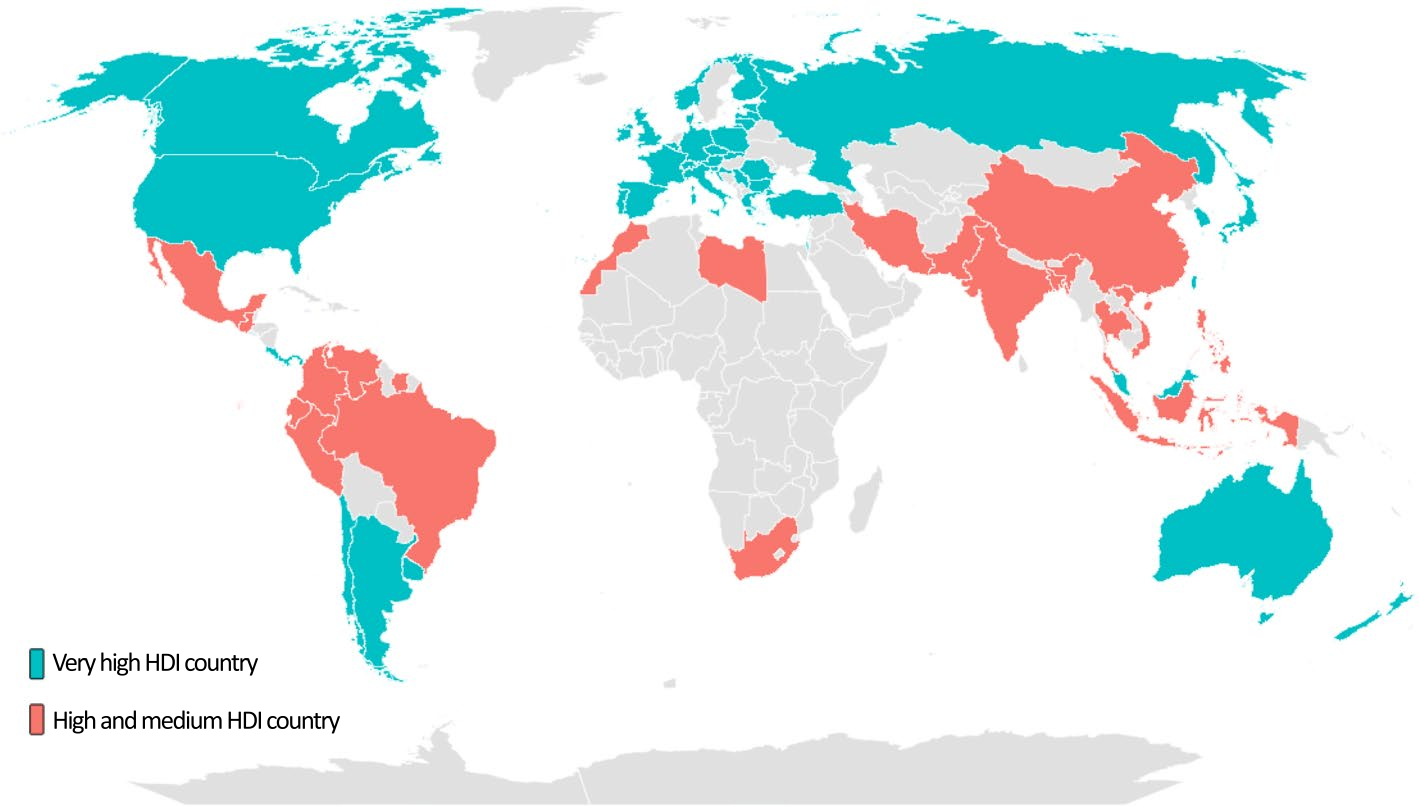
Geographical representation of the countries included in the analyses

Data from open source databases were used. First, Hofstede’s 6-dimensions cultural index was downloaded [28]; data for 70 countries with verified values of the indices was included. Second, data from the Global Burden of Diseases, Injuries, and Risk Factors Study (GBD) for year 2019 [33] was added to the dataset. Unfortunately, DALYs, YLD and YLL values were not available for one country; therefore, the final sample comprised 69 countries covering all the continents (Fig. 1). Finally, in an effort to take into account the socioeconomic development of a country, the Human Development Index (HDI) values from the United Nations Development Program database [34] were added to the dataset. No ethical approval was necessary as we did not use individual data.

### Study measures

Hofstede’s cultural dimensions are an empirically developed metric for measuring national culture using six dimensions. Scores for each dimension can range from 0–100. A higher score means a higher level of that particular dimension. Specifically, a higher score for Individualism means a more Individualism country (individuals in this country tend to take care of themselves and their immediate families only); for Power distance this means a more hierarchical society where people are more likely to accept a hierarchical order without any further justification. Masculinity means that the society places more emphasis on performance and work, and there is a higher differentiation between the genders; Uncertainty avoidance means a higher need to fight against uncertainty and a need for clarity, structure and rules. Long-term orientation means societies where people are future-oriented, adapt to circumstances (include tradition) and tend to save and invest; finally, Indulgence means a culture that puts more emphasis on leisure and satisfying one’s desires and where people feel happier and practice more sport [27, 28].

Population health was assessed using the Global Burden of Diseases, Injuries, and Risk Factors Study (GBD). In this study, the 2019 dataset, which included 369 diseases and injuries in 204 countries and territories, was used [35]. We used DALYs, YLD and YLL rates for all causes, both genders together and age standardised for each selected country. DALY is calculated as the sum of YLL and YLD. YLL, representing the number of years lost due to premature death, is calculated by multiplying the number of deaths by the remaining life expectancy in the country at the time of death. YLD, measuring the number of years lived with disability, is calculated by multiplying the number of incident cases by the average duration of disease to remission or death and the weight of disability [36]. Further, the GBD values for the three main risk factor groups (environmental, behavioural and metabolic) that affect DALYs, YLD and YLL were used. For more detailed insight, we also used separate DALYs, YLD and YLL values corresponding to the two most common conditions affecting them, i.e. cardiovascular diseases and neoplasms for DALYs and YLL, and musculoskeletal and mental disorders for YLD.

For stratification of countries by their socioeconomic development, the HDI from the United Nations Development Program database for year 2019 was used. The HDI is a summary measure of average performance on key dimensions of human development, specifically: life expectancy, knowledge and standard of living based on the Gross National Income per capita. Countries are usually divided into four ranks according to their HDI: very high HDI (≥ 0.800), high HDI (0.700–0.799), medium HDI (0.550–0.699), and low HDI (< 0.550) [34]. In the present study, there were no countries with a low HDI. Due to a low number of countries in the “high” and “medium” categories (*n* = 6 and *n* = 17, respectively), for the purpose of the analyses the countries were dichotomised according to their HDI into two categories: 1) very high HDI (labelled VH-HDI, *n* = 46), 2) high and medium HDI together (labelled HM-HDI, *n* = 23).

### Statistical analyses

For all statistical analyses, the JASP software, version 0.16.2 (JASP Team, University of Amsterdam, The Netherlands) was used. First, descriptive characteristics of Hofstede’s dimensions and the burden of disease, the main groups of risk factors and the most common causes of the burden of disease were evaluated. Due to the violation of the normality assumption and the different group sizes, the differences in means between the VH-HDI and the HM-HDI countries were assessed with the Bayesian version of the Mann–Whitney U test. The associations among Hofstede’s dimensions and the burden of disease, stratified by the dichotomised HDI, were evaluated using Bayesian correlation coefficients. In all tests performed, the evidence of support for each hypothesis was quantified by the Bayes Factor (BF), which was interpreted according to Lee and Wagenmakers [37]: BF > 100 Extreme evidence, BF 30–100 Very strong evidence, BF 10–30 Strong evidence, BF 3–10 Moderate evidence, BF 1–3 Anecdotal evidence, and BF = 1 No evidence.

## Results

Table 1 presents the characteristics of Hofstede’s dimensions stratified by the dichotomised HDI. In countries with VH-HDI, there was extreme evidence of a higher mean value of Individualism (BF = 120.9), moderate evidence of a lower mean value of Power distance (BF = 6.9) and a higher mean value of Long-term orientation (BF = 8.7).

**Table 1:** Descriptive characteristics of Hofstede’s dimensions, stratified by HDI

	VH-HDI				HM-HDI				
	Valid N	Missing	Mean	SD	Valid N	Missing	Mean	SD	BF
Power Distance	46	0	53.35	22.68	23	0	71.87	14.97	6.89
Individualism	46	0	52.02	22.67	23	0	26.91	15.47	120.90
Masculinity	46	0	45.65	21.25	23	0	53.74	12.05	0.60
Uncertainty Avoidance	46	0	69.80	23.00	23	0	63.00	22.13	0.39
Long-Term Orientation	44	2	54.73	21.41	19	4	33.95	20.73	8.74
Indulgence	43	3	47.63	18.94	19	4	51.90	28.70	0.33

*VH-HDI* Very high Human Development Index, *HM-HDI* High or medium Human Development Index, *SD* Standard Deviation, *BF* Bayes Factor assessing the Bayesian Mann–Whitney U test

Table 2 shows the descriptive characteristics of the burden of disease characterised by DALYs, YLD and YLL, stratified by the dichotomised HDI. Countries with VH-HDI had in general lower mean values of burden of disease. In terms of risk factor groups, there is a pronounced difference (BF > 100) between the VH-HDI and HM-HDI groups in metabolic risks and DALYs and YLL; environmental risks and DALYs, YLD and YLL; and behavioural risks and YLL. Countries with VH-HDI also had lower mean values of DALYs and YLL connected to cardiovascular diseases (with moderate to strong BF). On the other hand, these countries had higher mean values of DALYs and YLL connected to neoplasms, and YLD connected to musculoskeletal and mental disorders. However, the differences were supported with low to moderate evidence only.

**Table 2:** Descriptive statistics of the burden of disease, main groups of risk factors and most common causes of burden of disease, stratified by HDI

	VH-HDI		HM-HDI		
Burden of disease	Mean	SD	Mean	SD	BF
Valid N	46	46	23	23	
Total					
DALYs	21,280.6	3505.4	30,604.7	7055.5	1075.17
YLD	10,476.2	723.0	10,641.4	780.0	0.36
YLL	10,804.4	3527.7	19,963.3	6520.5	874.88
Metabolic risk factors					
DALYs	4169.1	1734.0	6737.6	1830.4	167.35
YLD	1123.6	204.5	1273.4	273.8	1.64
YLL	3045.5	1609.4	5464.3	1697.1	618.91
Environmental risk factors					
DALYs	1468.9	601.0	3747.0	2114.2	683.16
YLD	500.0	113.0	781.7	212.9	2431.90
YLL	968.9	510.7	2965.3	1929.7	320.97
Behavioural risk factors					
DALYs	5947.5	2010.9	8986.6	4250.9	21.79
YLD	1627.1	282.9	1561.0	446.7	0.52
YLL	4320.4	1867.4	7425.6	3846.5	133.92
Cardiovascular diseases					
DALYs	3264.1	1904.6	4790.0	1853.8	9.97
YLL	2916.3	1832.7	4427.7	1774.0	14.23
Neoplasms					
DALYs	3229.6	541.9	2839.0	489.1	5.76
YLL	3077.3	543.7	2768.4	479.5	1.99
Musculoskeletal disorders					
YLD	1968.9	354.4	1746.0	193.7	4.92
Mental disorders					
YLD	1770.4	323.2	1660.0	287.5	0.47

*VH-HDI* Very high Human Development Index, *HM-HDI* High or medium Human Development Index, *SD* Standard Deviation, There were no missing values, *BF* Bayes Factor assessing the Bayesian Mann–Whitney U test

**Fig. 2:**
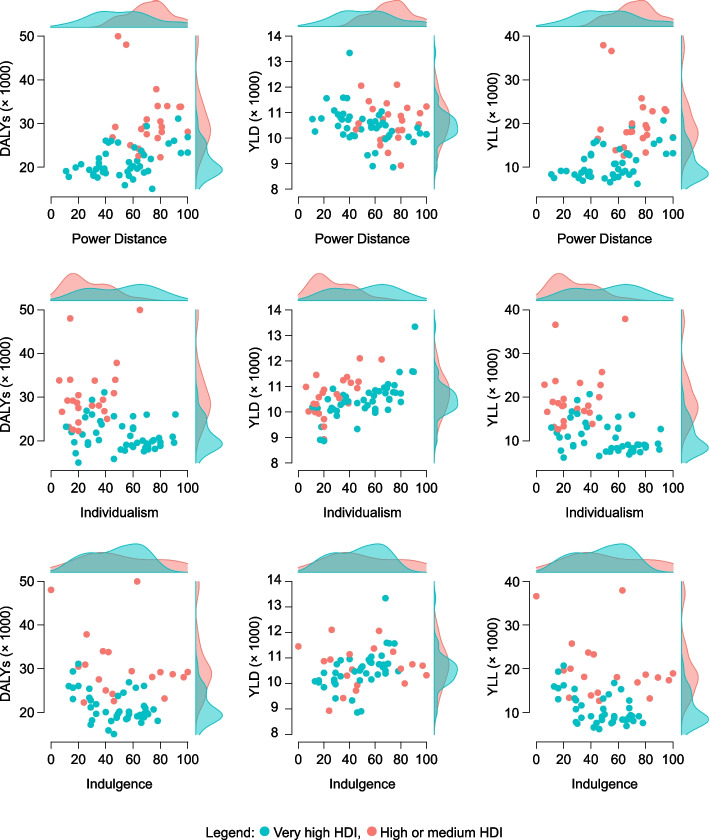
Scatter plots with marginal histograms depicting the relationship of Power distance, Individualism and Indulgence with the overall DALYs, YLD and YLL, stratified by the dichotomised HDI

To assess the associations among Hofstede’s dimensions and the burden of disease (DALYs, YLD, YLL) Bayesian correlation coefficients were evaluated, stratified by the dichotomised HDI. In countries with VH-HDI, we found at least strong evidence (BF > 10) of a positive correlation of Power distance with DALYs and YLL, Individualism and Indulgence with YLD, and a negative correlation of Long-term orientation with YLD and Indulgence with DALYs and YLL (see Table 3). In countries with HM-HDI, the only positive correlation with strong evidence was between Individualism and YLD. Based on the results in Table 3, further analyses of the associations between Power distance, Individualism and Indulgence with specific risk factors affecting the burden of disease were performed. The relationship between Power distance, Individualism and Indulgence with the overall DALYs, YLD and YLL, stratified by the dichotomised HDI, is depicted in Fig. 2.

**Table 3:** Bayesian correlation coefficients between Hofstede’s dimensions and the burden of disease, stratified by HDI

	VH-HDI (*n* = 46)				HM-HDI (*n* = 23)			
Hofstede's dimension & Burden of disease	r	Lower 95% CI	Upper 95% CI	BF	r	Lower 95% CI	Upper 95% CI	BF
Power Distance								
DALYs	0.448 *	0.172	0.641	20.6	−0.131	−0.488	0.279	0.3
YLD	−0.404	−0.609	−0.122	7.9	−0.045	−0.423	0.352	0.3
YLL	0.528 ***	0.268	0.698	180.3	−0.137	−0.492	0.275	0.3
Individualism								
DALYs	−0.197	−0.449	0.097	0.4	0.382	−0.039	0.660	1.2
YLD	0.667 ***	0.450	0.793	44,363.3	0.611 *	0.237	0.800	23.5
YLL	−0.332	−0.555	−0.043	2.2	0.340	−0.083	0.633	0.9
Masculinity								
DALYs	−0.124	−0.389	0.167	0.3	0.005	−0.385	0.392	0.3
YLD	0.135	−0.158	0.397	0.3	−0.013	−0.399	0.378	0.3
YLL	−0.151	−0.411	0.142	0.3	0.006	−0.383	0.393	0.3
Uncertainty Avoidance								
DALYs	0.219	−0.075	0.467	0.5	−0.031	−0.412	0.364	0.3
YLD	−0.200	−0.451	0.094	0.4	0.207	−0.212	0.543	0.4
YLL	0.259	−0.034	0.498	0.8	−0.058	−0.433	0.341	0.3
Long-Term Orientation								
DALYs	−0.065	−0.344	0.229	0.2	0.158	−0.297	0.536	0.3
YLD	−0.527 ***	−0.701	−0.260	129.5	−0.257	−0.602	0.209	0.5
YLL	0.044	−0.249	0.326	0.2	0.201	−0.260	0.565	0.4
Indulgence								
DALYs	−0.437 *	−0.639	−0.149	12.1	−0.280	−0.617	0.188	0.5
YLD	0.494 **	0.216	0.680	45.9	−0.031	−0.446	0.399	0.3
YLL	−0.537 ***	−0.709	−0.268	147.4	−0.299	−0.629	0.170	0.6

*VH-HDI* Very high Human Development Index, *HM-HDI* High or medium Human Development Index, *CI* Credible Interval, *DALYs* Disability-adjusted life years, *YLD* Years lived with disability, *YLL* Years of life lost, *BF* Bayes Factor: * BF > 10 (strong evidence), ** BF > 30 (very strong evidence), *** BF > 100 (extreme evidence)

Tables 4, 5 and 6 summarise the associations between Power distance, Individualism and Indulgence with burden of disease caused by specific risk factors and with specific causes of DALYs, YLD and YLL. In countries with HM-HDI, strong evidence of an association (with BF > 10) was found in a few relationships: Individualism was positively correlated with behavioural YLD, and Indulgence was positively correlated with metabolic YLD and negatively correlated with environmental DALYs, YLD and YLL. On the other hand, in countries with VH-HDI, Power distance and Indulgence were correlated with the majority of the indicators of burden of disease affected by specific risk factors. The correlations of Power distance with those indicators were positive, with the exception of mental disorders, while the correlations of Indulgence were negative, with the exception of musculoskeletal and mental disorders. There were a few correlations with strong evidence of an association (with BF > 10) in the relationships of Individualism: a negative correlation with the environmental risk factor DALYs and YLD, and a positive one with YLD affected by behavioural risk factors and mental disorders.

**Table 4:** Correlation (Bayesian correlation coefficients) between Hofstede’s Power distance, different groups of risk factors and the two most common causes of DALYs, YLD and YLL, stratified by the dichotomised HDI

	VH-HDI (*n* = 46)				HM-HDI (*n* = 23)			
Power distance & Burden of disease	r	Lower 95% CI	Upper 95% CI	BF	r	Lower 95% CI	Upper 95% CI	BF
Metabolic risk factors								
DALYs	0.540 ***	0.283	0.707	260.7	0.053	−0.346	0.429	0.3
YLD	0.400	0.118	0.606	7.3	0.017	−0.374	0.402	0.3
YLL	0.531 ***	0.272	0.700	195.5	0.054	−0.344	0.431	0.3
Environmental risk factors								
DALYs	0.604 ***	0.366	0.751	2650.8	0.059	−0.341	0.434	0.3
YLD	0.559 ***	0.307	0.720	490.2	0.198	−0.220	0.536	0.4
YLL	0.587 ***	0.344	0.740	1379.3	0.042	−0.354	0.421	0.3
Behavioral risk factors								
DALYs	0.433 *	0.155	0.630	14.6	−0.243	−0.568	0.178	0.5
YLD	−0.085	−0.356	0.204	0.2	−0.265	−0.582	0.158	0.5
YLL	0.479 **	0.209	0.664	44.9	−0.238	−0.564	0.183	0.5
Cardiovascular diseases								
DALYs	0.497 **	0.231	0.677	73.0	0.136	−0.276	0.491	0.3
YLL	0.493 **	0.226	0.674	65.1	0.134	−0.277	0.491	0.3
Neoplasms								
DALYs	0.284	−0.008	0.518	1.1	−0.255	−0.576	0.168	0.5
YLL	0.318	0.028	0.544	1.8	−0.247	−0.570	0.175	0.5
Musculoskeletal disorders								
YLD	−0.401	−0.607	−0.119	7.5	0.335	−0.088	0.630	0.8
Mental disorders								
YLD	−0.540 ***	−0.707	−0.283	264.2	−0.050	−0.427	0.348	0.3

*VH-HDI* Very high Human Development Index, *HM-HDI* High or medium Human Development Index, *CI* Credible Interval, *DALYs* Disability-adjusted life years, *YLD* Years lived with disability, *YLL* Years of life lost, *BF* Bayes Factor: * BF > 10 (strong evidence), ** BF > 30 (very strong evidence), *** BF > 100 (extreme evidence)

**Table 5:** Correlation (Bayesian correlation coefficients) between Hofstede’s Individualism, different groups of risk factors and the two most common causes of DALYs, YLD and YLL, stratified by the dichotomised HDI

	VH-HDI (*n* = 46)				HM-HDI (*n* = 23)			
Individualism & Burden of disease	r	Lower 95% CI	Upper 95% CI	BF	r	Lower 95% CI	Upper 95% CI	BF
Metabolic risk factors								
DALYs	−0.346	−0.566	−0.058	2.7	0.240	−0.181	0.566	0.5
YLD	−0.230	−0.476	0.063	0.6	0.216	−0.204	0.549	0.4
YLL	−0.344	−0.564	−0.055	2.6	0.224	−0.196	0.555	0.4
Environmental risk factors								
DALYs	−0.433 *	−0.630	−0.155	14.6	0.082	−0.321	0.452	0.3
YLD	−0.457 *	−0.648	−0.183	25.9	−0.116	−0.477	0.293	0.3
YLL	−0.408	−0.612	−0.127	8.6	0.103	−0.304	0.467	0.3
Behavioral risk factors								
DALYs	−0.123	−0.388	0.168	0.3	0.473	0.062	0.717	3.0
YLD	0.474 **	0.204	0.660	39.8	0.648 **	0.290	0.821	49.9
YLL	−0.205	−0.455	0.089	0.5	0.447	0.033	0.701	2.2
Cardiovascular diseases								
DALYs	−0.260	−0.499	0.033	0.8	0.244	−0.177	0.568	0.5
YLL	−0.262	−0.501	0.031	0.8	0.241	−0.181	0.566	0.5
Neoplasms								
DALYs	−0.053	−0.328	0.234	0.2	−0.117	−0.478	0.291	0.3
YLL	−0.102	−0.370	0.188	0.2	−0.112	−0.474	0.296	0.3
Musculoskeletal disorders								
YLD	0.355	0.067	0.572	3.1	−0.245	−0.569	0.177	0.5
Mental disorders								
YLD	0.544 ***	0.288	0.709	296.4	0.401	−0.019	0.672	1.4

*VH-HDI* Very high Human Development Index, *HM-HDI* High or medium Human Development Index, *CI* Credible Interval, *DALYs* Disability-adjusted life years, *YLD* Years lived with disability, *YLL* Years of life lost, *BF* Bayes Factor: * BF > 10 (strong evidence), ** BF > 30 (very strong evidence), *** BF > 100 (extreme evidence)

**Table 6:** Correlation (Bayesian correlation coefficients) between Hofstede’s Indulgence, different groups of risk factors, and the two most common causes of DALYs, YLD and YLL, stratified by the dichotomised HDI

	VH-HDI (*n* = 46)				HM-HDI (*n* = 23)			
Indulgence & Burden of disease	r	Lower 95% CI	Upper 95% CI	BF	r	Lower 95% CI	Upper 95% CI	BF
Metabolic risk factors								
DALYs	−0.624 ***	−0.769	−0.382	2994.7	−0.153	−0.532	0.301	0.3
YLD	−0.330	−0.560	−0.030	1.8	0.615 *	0.190	0.815	10.9
YLL	−0.631 ***	−0.773	−0.391	3992.9	−0.254	−0.600	0.212	0.5
Environmental risk factors								
DALYs	−0.474 *	−0.665	−0.192	28.0	−0.709 **	−0.866	−0.330	60.7
YLD	−0.401	−0.612	−0.107	5.9	−0.748 ***	−0.886	−0.395	152.7
YLL	−0.470 *	−0.662	−0.187	25.2	−0.694 **	−0.858	−0.306	43.7
Behavioral risk factors								
DALYs	−0.584 ***	−0.741	−0.328	660.2	−0.310	−0.636	0.159	0.6
YLD	−0.018	−0.306	0.275	0.2	−0.229	−0.584	0.235	0.4
YLL	−0.622 ***	−0.767	−0.379	2762.8	−0.316	−0.640	0.153	0.6
Cardiovascular diseases								
DALYs	−0.668 ***	−0.798	−0.442	20,181.9	−0.601	−0.807	−0.170	8.8
YLL	−0.667 ***	−0.797	−0.440	19,216.9	−0.593	−0.803	−0.159	7.9
Neoplasms								
DALYs	−0.443 *	−0.643	−0.156	13.8	−0.291	−0.624	0.178	0.6
YLL	−0.463 *	−0.657	−0.179	21.4	−0.309	−0.636	0.160	0.6
Musculoskeletal disorders								
YLD	0.624 ***	0.381	0.769	2978.2	0.076	−0.364	0.479	0.3
Mental disorders								
YLD	0.616 ***	0.371	0.763	2177.3	0.154	−0.300	0.533	0.3

*VH-HDI* Very high Human Development Index, *HM-HDI* High or medium Human Development Index, *CI* Credible Interval, *DALYs* Disability-adjusted life years, *YLD* Years lived with disability, *YLL* Years of life lost, *BF* Bayes Factor: * BF > 10 (strong evidence), ** BF > 30 (very strong evidence), *** BF > 100 (extreme evidence)

## Discussion

The aim of our study was to investigate whether national cultural characteristics, as expressed by Hofstede’s dimensions, have an impact on population health. To our knowledge, this is the first study providing an understanding of the associations of cultural characteristics to overall burden of disease, to three main group of risk factors affecting burden of disease and to the burden of disease caused by the most relevant groups of diseases. The findings show that, particularly in VH-HDI countries, cultural characteristics are associated with all three of the above-mentioned areas.

Regarding associations among Hofstede’s dimensions and the burden of disease we found that among countries with VH-HDI, a positive correlation with at least strong evidence was found between Power distance and the total DALYs and YLL, Individualism and YLD and Indulgence and YLD. A negative correlation with strong evidence was found between Long-term orientation and YLD as well as Indulgence and total DALYs and YLL. Within the group of countries with HM-HDI, a positive correlation with strong evidence was found only for Individualism and YLD. Hence, our results suggest that a higher the level of Individualism seems to lead to a higher number of YLD across all countries, regardless of the level of the HDI index itself, and therefore the level of development. In other words, a higher level of Individualism seems to be strongly associated with higher prevalence and duration of ill health in a society. This finding re-confirms the importance of social cohesion, equity and core principles of health promotion, as described earlier by Ottawa Charter for health promotion [38] and other studies [39–41].

We further found a strong correlation between Power distance and DALYs and YLL. We interpreted this finding as a higher level of centralisation, and a strong hierarchy seems to contribute to high premature mortality [42]. We further found a negative correlation with strong evidence between Indulgence and total DALYs and YLL, but positive with YLD. Thus, it seems from our findings that higher levels of Indulgence could extend life and reduce the overall burden of disease. This probably occurs through the positive effect of leasure time on our health [43] or more people being active in sports [44]. At the same time, higher levels of Indulgence are also associated with higher percentages of obese people in countries with enough food [27], which may explain the higher number of years with disabilities (e.g. obesity is associated with pain of various kinds [45].

Furthermore, in the examination of the Hofstede’s dimension in relation to different groups of risk factors (environmental, biological and behavioural risk) affecting DALYs, YLDs and YLL, we found that high values of Power distance are associated with all risk factors affecting DALYs, YLD and YLL. Our results shows, that a centralised and hierarchical society expressed through high values of Power distance seems to be a very important negative determinant for the impact of environmental and metabolic risk factors in loss of DALYs, YLL and YLD, though in the case of YLD and metabolic risk factors the correlation is with moderate evidence. In the case of environmental risk factors this can be interpreted with a low level of care for the surrounding life environment, rather expecting centralised action by governments or other hierarchically high authorities [46, 47]. The findings seem to signal that more individualist societies do care more about their environment, which is also suggested by the meta-analysis of Morren and Grinstein [48]. Further, Indulgence seems to be a very important positive determinant for the impact of all three groups of risk factors on loss of DALYs and YLL. This means that a higher level of Indulgence could save years of life. It has to be noted that these correlations were mostly found only within the group of VH-HDI countries. Among the HM-HDI countries we identified very few correlations; strong evidence between Indulgence and environmental risk factors for DALYs, YLL and YLD and a strong positive correlation between Individualism and behavioural risk factors. This difference between VH-HDI and MH-HDI could be related to high living standards and better access to the health care system [49].

Concerning the relationship of the Hofstede Index with the burden of disease caused by the most relevant groups of diseases we found no strong correlations between Hofstede’s dimensions and a selected groups of diseases among the HM-HDI countries. Among the VH-HDI countries, a higher level of Power distance was correlated to DALYs and YLL in the case of cardiovascular diseases. This correlation underlines the above-written considerations regarding distance to access of health care. A more decentralised and more accessible health care system saves lifes [42]. Individualism plays a negative role in the development of mental health problems in terms of loss of YLD; this underlines the negative role of a lack of social cohesion most likely leading to loneliness, which in turn leads to poorer mental health [50–52]. The most controversial correlations were identified in the case of Indulgence and the selected disease groups. A higher level of Indulgence seems to lead to the loss of a higher number of healthy life years; could Indulgence make us more tolerant in a negative sense to poor work conditions (often a proximal cause of musculoskeletal problems) or to negligence toward early identification of mental health problems? In an opposite way, a strong negative correlation between cardiovascular disease and neoplasms with Indulgence signals that a higher level of Indulgence could lead to fewer years of healthy life lost due to these diseases.

Regarding all the identified correlations, it is important to discuss what is the likely mechanism behind the influence of cultural characteristics on years of healthy life lost. The general pathway through well-established determinants of health and risk factors seems to be clear, and the most important group of determinants seems to be the environmental ones. However, our analysis strengthens the importance of synergism and the cumulative effect of the determinants of health [53]. Apparently, a cultural determinant is associated with behavior [54], and behaviour has an impact on the environment [55], resulting in a prevalence of risk factors in a specific population. For example, cultural practices influence dietary behaviour (e.g. mediterranean diet vs. american diet vs. central european diet etc.) and at the same time food choices have a profound impact not only on human health but also on the environment [56], which in turn influences health.

### Strength and limitations of the study

The strengths of our study include the use of the standardised GBD data for a comparison across locations on all continents. Another strength is that this is the first study to examine the relationship of cultural characteristics with health and opens up a potentially new area of research within population health research. It also provides valuable insights for future interventions in cultural determinants of health.

The study has several limitations. Our analysis is in principle a cross-sectional one and as such it disregards changes over time in both cultural characteristics and health outcomes. Taking into account increasing migration and multiculturalism in a recent globalised world, this might be perceived as a weakness. On the other hand, it can open the issue of more research on the single country level. Further, we did not employ any direct measure to assess the role of the health care system. We believe that on this level of analysis employment of the HDI is satisfactory, as there is a clear relation between HDI and the quality of a health system [49]. Another limitation could be the number of countries and the type of countries (e.g. low representation of the African continent and lack of representation of low-HDI countries) included in the analyses, which may weaken generalizability of our findings. The underrepresentation resulted from the limited number of countries for which Hofstede's index was available. Furthermore, due to having values of Hofstede’s dimensions for the whole population only, we did not analyse the correlations by sex. However, based on finding that masculinity as a Hofstede dimension did not show a significant correlation with any of the burden of disease parameters, this does not seem to be a major limitation in our work. In addition, although steps were taken to identify and control for confounders, there may be additional potential confounders that may have influenced the associations observed in our analyses. Last but not least, the complexity of the considerations in the paper can also be a limitation. This complexity of the considerations arises from the fact that all variables in the dataset are aggregated variables, which themselves carry complex information.

### Implications and recommendations

The results of this research have practical relevance and provide valuable insights for health professionals, policymakers, and educators aiming to improve health outcomes. This study underlines the importance of considering cultural factors in healthcare planning and policy-making, suggesting that a culturally informed approach could significantly impact health promotion and disease prevention. For example, societies with high levels of individualism, health promotion efforts could focus on strengthening community ties and enhancing social support systems to address the negative effects of social isolation. Furthermore, public health authorities of countries with low levels of Indulgence could focus more on encouraging people to be careful about their leisure time and sufficient physical activity. This could mitigate the negative effects of low levels of Indulgence on the burden of disease. Moreover, countries with high power distance, where hierarchical structures may restrict access to healthcare, decentralizing health services could reduce barriers to access them, leading to lower premature mortality and improved health equity. In terms of education, we recommend greater interconnection between the sciences and the humanities to ensure a holistic view of human health.

Furthermore, this research lays a strong foundation for future studies. Longitudinal research is necessary to better understand the causal relationships between cultural characteristics and health outcomes. Additionally, exploring the mechanisms through which cultural characteristics influence health and examining the role of mediators and moderators in these relationships could lead to more effective, personalized health interventions. It would also be useful to explore the association of cultural characteristics with specific types of disease (e.g. lung disease, heart disease) or in different populations of interest (e.g. with respect to specific lifestyle or health problems).

## Conclusion

In conclusion, our study clearly shows the correlation of some of Hofstede’s dimensions (in specific, Individualism, Power distance and Indulgence) to the overall burden of disease characteristics. We found these correlations to be more frequent in very high HDI countries. Individualism was associated with more years lived with disability (YLD), highlighting the importance of social cohesion in health promotion. Power distance correlated with higher premature mortality (YLL), reflecting the negative impact of hierarchical societal structures on access to healthcare. Indulgence showed mixed outcomes, extending life but increasing the years lived with disability. The amount of YLD’s is often influenced by both early detection and good management of disease, so further analysis of role of individualism and indulgence on YLD’s would be relevant. The underlying significance of our findings is that interventions expected to save health life years must take into account acultural characteristics of the health of target populations. If confirmed longitudinally, adapting policies to these factors can enhance their effectiveness and sustainability.

## Data Availability

Data is available online in open databases.

## Abbreviations

BF: Bayes factor
DALYs: Disability-adjusted life years
GBD: Global Burden of Diseases
HDI: Human Development Index
HM-HDI: High and medium HDI country
QALY: Quality adjusted life years
VH-HDI: Very high HDI country
YLD: Years lived with disabilities
YLL: Years of life lost
